# Sex-Specific Microglial Activation and SARS-CoV-2 Receptor Expression Induced by Chronic Unpredictable Stress

**DOI:** 10.3389/fncel.2021.750373

**Published:** 2021-11-24

**Authors:** Ling Yan, Mohan Jayaram, Keerthana Chithanathan, Alexander Zharkovsky, Li Tian

**Affiliations:** ^1^Institute of Biomedicine and Translational Medicine, Department of Physiology, Faculty of Medicine, University of Tartu, Tartu, Estonia; ^2^Institute of Biomedicine and Translational Medicine, Department of Pharmacology, Faculty of Medicine, University of Tartu, Tartu, Estonia

**Keywords:** chronic unpredictable mild stress, neuroinflammation, glial cells, COVID-19, SARS-CoV-2, neuropsychiatric disorders

## Abstract

The coronavirus disease 2019 (COVID-19) pandemic has generated a lot of stress and anxiety among not only infected patients but also the general population across the globe, which disturbs cerebral immune homeostasis and potentially exacerbates the SARS-CoV-2 virus-induced neuroinflammation, especially among people susceptible to neuropsychiatric disorders. Here, we used a chronic unpredictable mild stress (CUMS) mouse model to study its effects on glia-mediated neuroinflammation and expression of SARS-CoV2 viral receptors. We observed that female mice showed depressive-like behavior after CUMS, whereas male mice showed enhanced anxiety and social withdrawal. Interestingly, CUMS led to increased amounts of total and MHCII^+^ microglia in the hippocampi of female mice but not male mice. mRNA levels of SARS-CoV-2 viral receptors angiotensin-converting enzyme 2 (*Ace2*) and basigin (*Bsg*) were also upregulated in the prefrontal cortices of stressed female mice but not male mice. Similarly, sex-specific changes in SARS-CoV-2 viral receptors *FURIN* and neuropilin-1 (*NRP1*) were also observed in monocytes of human caregivers enduring chronic stress. Our findings provided evidence on detrimental effects of chronic stress on the brain and behavior and implied potential sex-dependent susceptibility to SARS-CoV-2 infection after chronic stress.

## Introduction

The coronavirus disease 2019 (COVID-19) pandemic caused by the severe acute respiratory syndrome coronavirus 2 (SARS-CoV-2) has been giving a significant psychosocial impact on human beings leading to our prolonged state of mental stress (Saladino et al., 2020). Recent surveys have suggested that not only children and young adults (Orgilés et al., 2020) but also health caregivers and aging people (Iodice et al., 2021) are at high risk of developing anxious symptoms and COVID-19 comorbidities, which include neurological diseases and psychiatric disorders (Kempuraj et al., 2020; Nuzzo and Picone, 2020). Furthermore, psychiatric patients were also reported to have higher COVID-19 mortality than common patients (Lega et al., 2021; Vai et al., 2021). Notably, women are suggested to be mentally and behaviorally more aware of the pandemic as a serious health problem (Bwire, 2020; Galasso et al., 2020) and more susceptible to disease-related anxiety and depression (Hou et al., 2020; Thibaut and van Wijngaarden-Cremers, 2020) but in contrary male COVID-19 patients have more severe symptoms and outcomes (Huang et al., 2020; Jin et al., 2020; Peckham et al., 2020; Vahidy et al., 2021). Considering the huge population being directly or indirectly affected by waves of the pandemic globally, the detrimental effect of psychosocial stress on mental health during and after the ongoing pandemic should not be neglected in the society.

The main host cell receptor for the SARS-CoV-2 virus is angiotensin-converting enzyme 2 (*ACE2*) (Hoffmann et al., 2020; Yang et al., 2020). Another associated viral receptor is basic immunoglobulin (*Basigin, BSG*) (Wang et al., 2020), which is coregulated by viral infection together with *ACE2* and modulates *ACE2* abundance. In addition, neuropilin-1 (*NRP1*) and *Furin* also assist *ACE2* in viral attachment (Xia et al., 2020). These viral receptors are expressed by different cell types including brain cells (Fodoulian et al., 2020; Vargas et al., 2020; Chen et al., 2021) and can be regulated by neurological pathologies (Davies et al., 2020; Fodoulian et al., 2020; Qiao et al., 2020). The cerebral entry routes of the virus are suggested to possibly include the vasculature, the olfactory and trigeminal nerves, the cerebrospinal fluid, and the lymphatic system (Desforges et al., 2019; Boldrini et al., 2021).

Glia-induced neuroinflammation is a common pathological observation in neurological cases of COVID-19 (Tremblay et al., 2020; Vargas et al., 2020), as microglia and astrocytes are key pathogen-combating players in the brain during SARS-CoV-2 infection (McMahon et al., 2021; Yang et al., 2021). However, when primed by stress, they can cause serious neurological and psychiatric complications (Tay et al., 2017; Madore et al., 2020). So far, the stress effect on COVID-19-associated neuropathology and the underlying glia-mediated mechanisms have not been reported. To address this question, we hereby used a chronic unpredictable mild stress (CUMS) mouse model aligned with a human monocyte transcriptomic dataset published by a previous study on human subjects who endured stress caused by caregiving of their family members (Miller et al., 2014). We found that the abundancy of microglia, especially MHCII^+^ microglia in the hippocampus and mRNA levels of *Ace2* and *Bsg* in the prefrontal cortex (PFC), was upregulated only in stressed female mice but not in males. Similarly, viral receptor *FURIN* was also increased in monocytes of human female caregivers enduring chronic stress, whereas *NRP1* was decreased in stressed male caregivers.

## Materials and Methods

### Animals

Wild-type C57BL/6NTac female and male mice (3 months old) were housed and bred in the laboratory animal facility at the Institute of Biomedicine and Translational Medicine, University of Tartu. Mice of the same sex from different litters were housed in the same 1264C Euro standard type II cages (Tecniplast, West Chester, PA, United States) measuring 268 mm × 215 mm × 141 mm. Cages contained aspen chips for bedding and aspen wool for nesting material, which were replaced once a week. Mice were kept under standard conditions with unlimited access to food and water on a 12/12 h light/dark cycle (light on during 07:00–19:00). All animal procedures in this study were performed in accordance with the European Communities Directive with license No. 171 (July 1, 2020) issued from the Estonian National Board of Animal Experiments.

### Chronic Unpredictable Mild Stress Procedure

**FIGURE 1:**
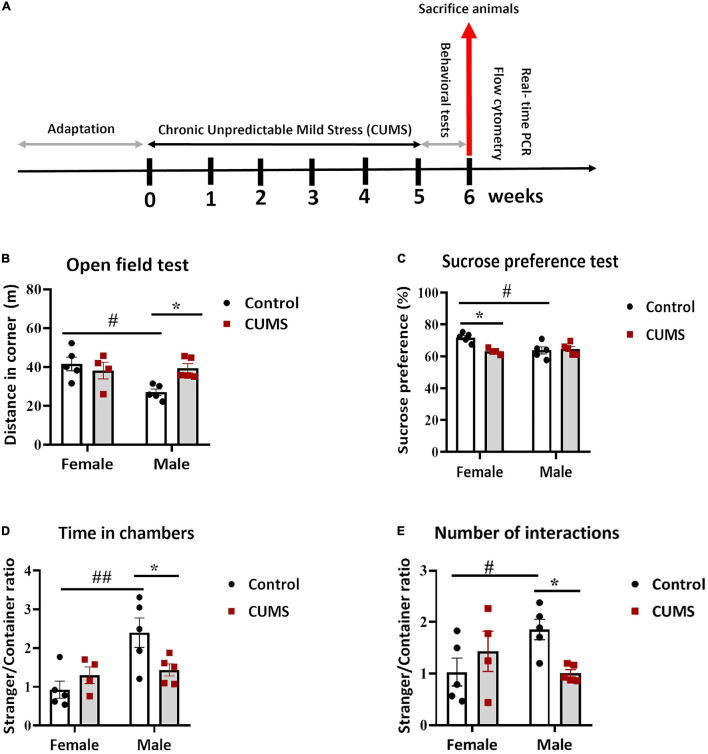
Chronic unpredictable mild stress (CUMS) induced depressive-like behavior in female mice and anxious and social withdrawal in male mice. **(A)** A schema representing experimental design. **(B)** In the open field test, control male mice traveled less in the corners than control female mice, whereas CUMS males traveled more than control male mice. **(C)** Control female mice consumed more sucrose than control male mice, whereas CUMS female mice consumed less than control female mice. **(D,E)**. In the three chamber sociability test, control male mice were more social than control female mice, in terms of ratios of the time spent in a chamber with a stranger mouse vs. that with an empty container **(D)** and the number of interactions with the stranger mouse vs. the empty container **(E)**, respectively, whereas CUMS male mice showed less sociability than control male mice. The symbols * and # represent CUMS and sex differences, respectively (two-way ANOVA). *p < 0.05, **p < 0.01, and #*p* < 0.05.

Mice from each litter were randomly assigned into two groups: Control and CUMS group, *n* = 5 per group. One female from the CUMS group died during the experiment. We used the resource equation to calculate the sample size used for the present study (Mead, 1988). The scheme of experimental design is shown in Figure 1A. The CUMS procedure was adapted from Liu et al. (2019). Briefly, after a week of transfer adaptation, mice were daily subjected to one of seven CUMS stressors for five consecutive weeks including: food and water deprivation overnight, rat odor and isolation overnight, restraint in 50-ml tube for 2 h, wet bedding and tilted cage, stroboscopic illumination overnight, flipped light/dark exposure, and swimming at 18°C for 10 min. All stressors were applied individually and were randomly scheduled at different time points per day, and no single stressor was applied for two consecutive days to sustain an unpredictable procedure. Following the CUMS procedure, the same mice were used for behavioral tasks, flow cytometry, and real-time quantitative PCR (RT-qPCR).

### Open Field Test

Before experimentation, mice were habituated to room light for 1 h. Locomotor and exploratory activities of individual mouse were measured for 30 min in a box (44.8 cm × 44.8 cm × 45 cm) connected to a computer, which registered the distance traveled, number of rearing, corner visits, distance in corner, and time spent in the central part of the box. Data were recorded by a behavioral analytic system (Technical and Scientific Equipment GmbH, Bad Homburg Germany). The floor of the box was cleaned with 70% ethanol and dried thoroughly after each mouse.

### Sucrose Preference Test

Mice were single caged, and two bottles of fresh water were kept for adaptation for 3 days. On the 4th day, one bottle was replaced with 1% sucrose-containing water. The two bottles were weighed before and 12 h afterward. Sucrose preference (SP) was calculated according to the following formula, SP = sucrose intake/(sucrose intake + water intake) × 100%.

### Three-Chamber Test

A rectangular three-chamber box made from clear Plexiglas was divided into an open middle section and two other identical side sections, which each accommodated a lid-covered and wire-structured cup-like container large enough to enclose a single mouse, allowing free exchange of air but not direct physical contacts between the mice on either side of it. A test mouse was first habituated in the central chamber for 5 min, then introduced to a stranger-1 mouse located in a container for the sociability test. Mice were left to freely explore the three chambers for the next 10 min. All stranger mice of both sexes were at the same age as test mice and habituated to the apparatus during the previous day (30 min habituation for three times). The box and wire containers were cleaned with 70% ethanol and dried thoroughly after each test. Social exploration was defined as time spent in each chamber and social interaction as number of contacts when the head and front paws of a test mouse were within 3 cm vicinity of the container wall as recorded by a camera (Noldus, Wageningen, Netherlands). The ratios of time spent with a stranger mouse or in an empty chamber and number of contacts with a stranger mouse or in an empty container were calculated as sociability indices, respectively.

### Flow Cytometry

Mice were euthanized with CO_2_. Dissected hippocampi were gently homogenized through 70 μm cell strainers (#352350, BD Bioscience, San Jose, CA, United States) in ice-cold phosphate-buffered saline (PBS) with 1% fetal calf serum. Homogenates were washed and centrifuged at 500 × *g* for 5 min. Isolated cells were blocked with 10% rat serum in ice-cold PBS for 1 h. Brain cells were stained with 0.5 μl anti-mouse MHCII-Brilliant Violet (BV) 711 (#107643, BioLegend, San Diego, CA, United States), CD11b-BV 421 (#101251, BioLegend, San Diego, CA, United States), CD45-BV 650 (#103151, BioLegend, San Diego, CA, United States), Glast-APC (#130-123-555, Miltenyi Biotech, Bergisch Gladbach, Germany), and O4-PE (#130-117-357, Miltenyi Biotech, Bergisch Gladbach, Germany) with the corresponding isotype control antibodies (all from BioLegend, San Diego, CA, United States): rat IgG2b-BV711 (#400653), IgG2b-BV421 (#400639), IgG2b-BV650 (#400651), IgG2b-APC (#400219), and IgM-PE (#401611) in PBS with 1% fetal calf serum on ice for 1 h. Cells were washed, resuspended in 500 ml PBS and acquired with a Fortessa flow cytometer (BD Bioscience, San Jose, CA, United States). Data were analyzed by Kaluza v2.1 software (Beckman Coulter, Indianapolis, IN, United States). Astrocytes were defined as Glast^+^ cells, oligodendrocyte precursor cells (OPCs) as O4^+^ cells, microglia as CD45^low^CD11b^hi^ and MHCII^+^ cells. Cell populations were calculated as the percentages among total brain cells or among microglia.

### Total RNA Isolation and RT-qPCR

Total RNAs were extracted from the PFC tissue by TRIzol (Molecular Research Center, Cincinnati, OH, United States) and reversely transcribed with a RevertAid First Strand cDNA Synthesis Kit (Thermo Fisher Scientific, Waitham, MA, United States). RT-QPCR was performed by using corresponding primers and 5 × HOT FIREPol^®^ EvaGreen^®^ qPCR Supermix (Solis BioDyne, Tartu, Estonia) on a PCR instrument equipped with QuantStudio 12KFlex Software v.1.2.2 (Thermo Fisher Scientific, Waitham, MA, United States) according to the instructions of the respective manufacturers. The primers (Ma et al., 2020) listed in Supplementary Table 1 were purchased from TAG Copenhagen A/S (Frederiksberg, Denmark). Quantification was performed by normalizing Ct values of target genes to that of the reference gene (β-actin) and expressed as exponential fold changes against the average ΔCt value of the control group (2^–ΔΔCt^).

### Human and Mouse Gene Expression Analysis

Microarray dataset of a human stress study on monocytes (GSE52319) was retrieved from Gene Expression Omnibus (GEO) and analyzed by GEO2R therein for log values of fold changes (Log2FC) and adjusted *p*-values of differential expression. Identified target genes were counterchecked in various brain RNAseq databases (Supplementary Figures 2–5). To analyze spatial distributions of *Ace, Ace2, Nrp1, Nrp2, Bsg*, and *Furin*, images of Nissl and *in situ* hybridization (ISH) staining in the mouse hippocampus and prefrontal cortical areas were downloaded from Allen Mouse Brain Atlas database^1^ with their permission (Lein et al., 2007; Bicks et al., 2015), and the Brain explorer^®^ 2 software (Sunkin et al., 2013) was used to closely visualize and quantify gene expression levels based on staining intensity in ISH images.

### Statistical Analysis

GraphPad 8.0.1 (San Diego, CA, United States) was used for statistical analyses and graphical presentations. Two-way ANOVA was used to evaluate the interaction effect between CUMS and sex and their main effects, with Tukey’s test used for *post hoc* multiple comparisons only if significant interaction effects were found. Statistical significance was set at *p* < 0.05, and data were reported as mean ± SEM.

## Results

### Chronic Unpredictable Mild Stress Induces Depressive-Like Phenotype in Female Mice but Anxiety and Social Withdrawal in Male Mice

To assess the impact of CUMS procedure (experimental design shown in Figure 1A), female and male C57Bl/6N mice were evaluated behaviorally. Bodyweights did not show any significant CUMS and sex interaction or CUMS main effect on both female and male mice during the 5 weeks of CUMS (Supplementary Figure 1A). In the open field test, there was a significant interaction between CUMS and sex in distance moved in corners [*F* (1, 15) = 6.35, *p* < 0.05], showing that only male mice traveled more distances in corners after CUMS, whereas a lower level in control males compared to control females was also observed (Figure 1B). Meanwhile, no significant changes in total locomotor activities were observed in either sex or treatment groups (Supplementary Figure 1B). In addition, although no interaction effect was seen, there was a significant main effect of stress in the ratio of time spent in corners vs. center [*F* (1, 15) = 7.29, *p* < 0.05], and male mice had more such a trend (Supplementary Figure 1C). These results indicated increased anxiety in male mice after CUMS. To further assess anhedonic behavior, we performed the sucrose preference test. A significant interaction between CUMS and sex in sucrose preference was found [*F* (1, 15) = 8.09, *p* < 0.05], showing that female mice had significant reduction in sucrose preference, hence showing anhedonia, after CUMS as compared to their control counterparts, whereas male mice did not respond to CUMS but only control male mice showed less sucrose preference compared to control female mice (Figure 1C). Interaction between CUMS and sex was also noticed in the three-chamber sociability test, showing significances in the ratio of time spent in the social chamber vs. the empty container chamber [*F* (1, 15) = 6.43, *p* < 0.05] (Figure 1D) and the ratio of a number of interactions with a stranger mouse vs. an empty container [*F* (1, 15) = 6.65, *p* < 0.05] (Figure 1E), respectively. Specifically, control male mice showed more social time and interactions compared to control female mice, both of which were dampened by CUMS in male mice, whereas no CUMS effect was found in female mice (Figures 1D,E).

### Chronic Unpredictable Mild Stress Induces Microglial Activation in the Hippocampi of Female but Not Male Mice

**FIGURE 2:**
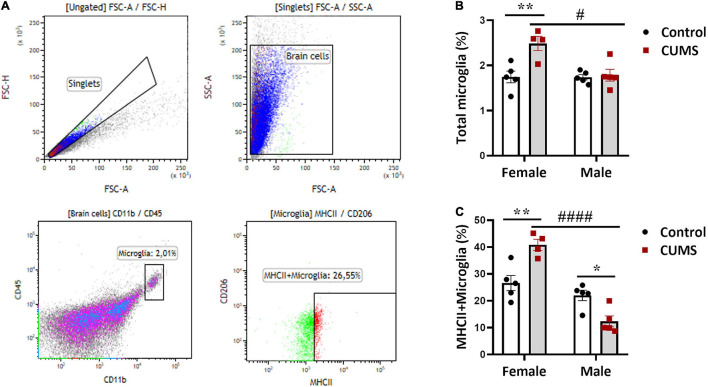
Hippocampal microglia were activated in CUMS female mice. **(A)** Representative graphs showing gating strategy for astrocytes, OPCs, microglia, and MHCII^+^ microglia; **(B)** percentage of microglia among total brain cells present in the hippocampus; **(C)** percentage of MHCII^+^ microglia among total microglia. The symbols * and # represent CUMS and sex differences, respectively (two-way ANOVA). *p < 0.05, **p < 0.01, #*p* < 0.05, and ####*p* < 0.0001.

To understand CUMS-induced neuroinflammation, we first used flow cytometry to quantitate astrocytes, OPCs, and microglia. Gating strategy was depicted in representative dotplots in Figure 2A and Supplementary Figure 2B, and isotype antibody stainings used as negative controls in the experiment were shown in Supplementary Figure 2A. Significant interactions between CUMS and sex were found in the percentages of total microglia [*F* (1, 15) = 8.22, *p* < 0.05] and MHCII^+^ subtype of microglia [*F* (1, 15) = 27.27, *p* < 0.001]. In female mice, the percentages of total microglia (Figure 2B) and MHCII^+^ microglia (Figure 2C) in CUMS group were both increased compared to controls, indicating stress-induced neuroinflammation in female mice. By contrast, there was a decrease in MHCII^+^ microglia in CUMS male mice compared to controls (Figure 2C), implying immunosuppressive effect of CUMS in male mice. We noticed no significant interaction effects between CUMS and sex on astrocytes and OPCs. However, main sex and stress effects were observed on astrocytes [*F* (1, 15) = 8.5, *p* < 0.01] and OPCs [*F* (1, 15) = 9.44, *p* < 0.01], respectively. Astrocytes were trendily higher in control male mice compared to control female mice (Supplementary Figure 2C), and OPCs were trendily decreased in CUMS male mice compared to control male mice (Supplementary Figure 2D).

### Spatial and Cellular Distributions of SARS-CoV-2 Receptor Genes in the Mouse and Human Brains

**FIGURE 3:**
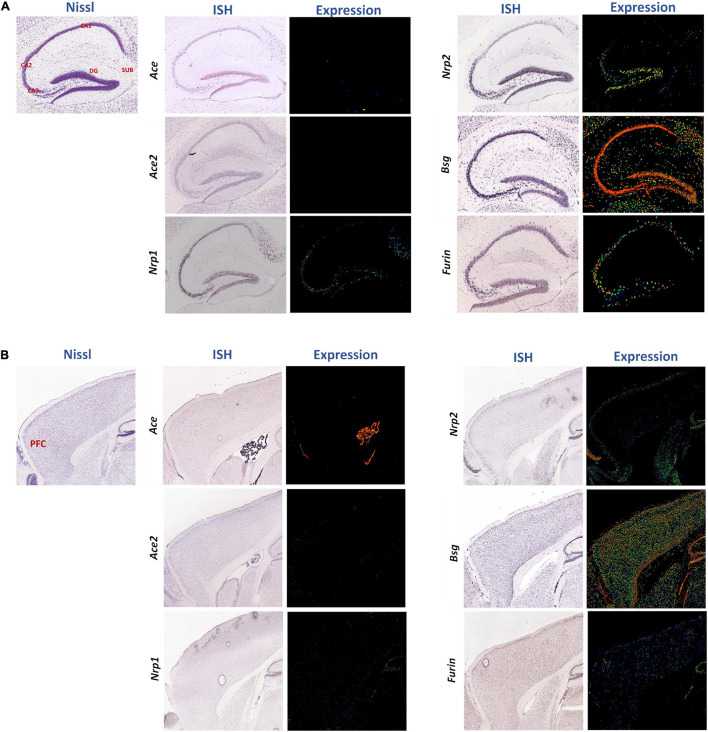
Spatial distributions of the SARS-CoV-2 receptors in the sagittal sections of mouse hippocampus and PFC region. Spatial distributions of the SARS-CoV-2 receptors *Ace*, *Ace2*, *Nrp1*, *Nrp2*, *Bsg*, and *Furin* in the mouse hippocampus **(A)** and cerebral cortex region **(B)** were studied. In representative Nissl images, the cornu ammonis (CA)1, CA2, CA3, dentate gyrus (DG), and subiculum (SUB), and PFC are labeled in red. The middle and right columns represent ISH and pseudo-colored expression images, respectively. Blue, green, and red pseudo-colors encode staining intensities (low, medium, and high) in expression images, respectively. All images were downloaded from the Allen Mouse Brain Atlas (©2004 Allen Institute for Brain Science) (Lein et al., 2007).

Furthermore, we analyzed spatial distributions of the SARS-CoV-2 receptors in the normal mouse brain using the ISH data in the Allen Mouse Brain Atlas covering the hippocampus (Figure 3A) and the forebrain (Figure 3B, the PFC marked in red). Expressions of most of these receptors were observed in both regions, especially *Bsg*, *Nrp2*, and *Furin* (Figures 3A,B), whereas *Ace* and *Ace2* levels were low in the parenchyma (except for high level of *Ace* in the choroid plexus in Figure 3B) and *Nrp1* was expressed intermediately in the hippocampus (Figure 3A). Using the Brain explorer^®^ 2 software, we further scrutinized the spatial distributions of these genes within the PFC areas, namely the anterior cingulate cortex, prelimbic area, infralimbic area, and orbital area, and we found their overall enrichment in the orbital area except *Ace2*, as summarized in Supplementary Table 2. Gene expressions of these receptors were also checked in the human PFC using a human protein atlas dataset, also showing that *ACE2* had very low expression, whereas the other genes were expressed more abundantly but with low regional specificity (Supplementary Figure 3).

As we were interested of glial cell expression of these genes, we further explored the Stanford brain RNAseq database (Zhang et al., 2014), showing they were enriched in different brain cell types in the human and mouse brains (Supplementary Figure 4). Specifically, *Ace* was expressed most abundantly in OPCs in mice but not detectable in human glial cells. *Ace2* was enriched in endothelia but not present in any glial cells in humans and mice. *Bsg* was present not only in human and mouse endothelia but also in human astrocytes. *Furin* was highly expressed in microglia in mice and astrocytes in humans. *Nrp1* was present mainly in endothelia in mice but in astrocytes and microglia in humans, whereas *Nrp2* was enriched in both human and mouse microglia. Furthermore, among these genes, *Ace* and *Nrp2* were expressed in mouse neurons. We also explored the mouse vascular single-cell transcriptomic database from Belsholtz lab (He et al., 2018; Supplementary Figure 5). *Ace*, *Ace2*, *and Bsg* were not enriched in any glial types compared to the other cell types studied there, with *Ace and Bsg* well enriched in endothelial cells and *Ace2* in smooth muscle cells. *Furin* was expressed broadly including astrocytes, whereas *Nrp1* and *Nrp2* were enriched not only in endothelial cells and fibroblasts but also present in microglia and astrocytes. Exploring yet another single-cell transcriptomic database on the mouse PFC (Bhattacherjee et al., 2019), we observed that all genes were enriched in endothelia. Meanwhile, *Nrp1, Bsg*, and *Furin* were all enriched in astrocytes, *Nrp1, Nrp2*, *Bsg*, *and Furin* in microglia, *Bsg* in oligodendrocytes and OPCs, whereas *Ace*, *Nrp1*, *Nrp2*, and *Bsg* in neurons as well (Supplementary Figure 6). In summary, a consensus result of these public datasets suggests that *Ace*, *Ace2*, and *Bsg* were enriched in vascular endothelia and associated smooth muscle cells, besides neurons, whereas *Nrp1, Nrp2*, *Bsg*, *and Furin* were expressed by glial cell types.

### Chronic Unpredictable Mild Stress Induces Gene Expressions of SARS-CoV-2 Receptors in the Prefrontal Cortex of Female but Not Male Mice

**FIGURE 4:**
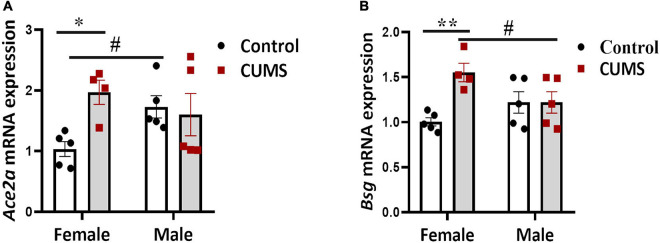
SARS-CoV-2 receptors were upregulated in the PFC of female mice. Sex-dependent upregulations of *Ace2a* **(A)** and *Bsg* **(B)** were detected using RT-qPCR. The symbols * and # represent CUMS and sex differences, respectively (two-way ANOVA). *p < 0.05, **p < 0.01, and #*p* < 0.05.

We next quantified gene expressions of the viral receptors and some pro-inflammation cytokines in the PFC of CUMS and control mice. Significant interaction effects between CUMS and sex on the expressions of *Ace2a* [*F* (1, 15) = 5.17, *p* < 0.05] and *Bsg* [*F* (1, 15) = 7.15, *p* < 0.05] were seen, implying a possible sex-specific effect of CUMS on potentiating viral entry and/or spreading in the brain. Specifically, *Ace2a* (Figure 4A) and *Bsg* (Figure 4B) were upregulated only in female mice but not in male mice after CUMS, whereas enhanced level of *Ace2a* (Figure 4A) in control male mice and suppressed level of *Bsg* (Figure 4B) in CUMS male mice, respectively, were seen compared to female mice. No interaction effects between CUMS and sex on other viral receptors and cytokines were found. However, main stress and sex effects were observed on *Ace2b* [*F* (1, 15) = 9.37, *p* < 0.01] and *Nrp1* [*F* (1, 15) = 8.0, *p* < 0.05], respectively. A trend of enhancing effect of CUMS on *Ace2b* was seen in female mice (Supplementary Figure 7B), and *Nrp1* had trendily higher basal level in control male mice compared to control female mice (Supplementary Figure 7C). Furthermore, cytokine *Il7* showed CUMS main effect too [*F* (1, 15) = 11.77, *p* < 0.01] and was trendily dampened in CUMS male mice compared to control male mice (Supplementary Figure 7G), whereas cytokines *TNF* and *Il1b* showed no significant changes in both female and male mice (Supplementary Figures 7F,H).

### Chronic Stress Induces Changes in Viral Receptor Gene Expressions in Human Monocytes

To further characterize stress effect on viral receptors and immune responses in human context, we searched GEO and discovered a dataset (GSE52319) that studied the influence of chronic stress, caused by caregiving to patients, on signaling pathways that regulate inflammation in human monocytes. This study found increased expression of genes bearing response elements for nuclear-factor kappa B, a key pro-inflammatory transcription factor among chronically stressed (CS) caregivers compared to non-stressed (NS) subjects. Simultaneously, CS caregivers showed reduced expression of genes with response elements for the glucocorticoid receptor, a transcription factor that conveys cortisol’s anti-inflammatory signals to monocytes (Miller et al., 2014). Their transcriptomic result revealed that *NRP1* was downregulated in stressed male caregivers, whereas a significant upregulation of *FURIN* was observed in stressed female caregivers (Table 1).

**TABLE 1:** Expressions of *ACE, ACE2, NRP1, NRP2, BSG*, and *FURIN* in monocytes of human subjects enduring chronic stress (CS) as compared to non-stressed (NS) controls.

Gender	Gene symbol	Adj. *p-*value	Log2FC
Females (CS vs. NS)	*ACE*	9.41E–01	−1.76E–03
	*ACE2*	2.23E–01	2.80E–02
	*NRP1*	4.18E–01	−5.45E–02
	*NRP2*	3.66E–01	1.97E–01
	*BSG*	8.47E–01	−2.67E–02
	***FURIN***	**2.32E–04**	**1.97E–01**
Males (CS vs. NS)	*ACE*	2,37E–01	1,65E–02
	*ACE2*	7,45E–01	−1,27E–02
	***NRP1***	**2,61E–02**	−**1,07E–01**
	*NRP2*	1,38E–01	2,40E–01
	*BSG*	3,35E–01	1,35E–01
	*FURIN*	3,09E–01	9,86E–02

*Data retrieved from GEO dataset GSE52319 (Miller et al., 2014); male mice: CS (n = 44) vs. NS (n = 60); female mice: CS (n = 57) vs. NS (n = 100). Bold text indicates statistically significant differences.*

## Discussion

Since there is still a knowledge gap on the relationship between psychosocial stress and COVID-19 disease development, the present study aims to investigate whether there is an exacerbating effect of chronic stress on glial activation that may contribute to SARS-CoV-2-induced brain infection and pathology. Our most interesting finding was sex-dependent linkage of chronic stress to anxiety- and depressive-related behaviors, microglial activation, and expression of SARS-CoV-2 receptors.

We first used a CUMS mouse model showing its behavioral effects in both female and male mice. Here, we found that female CUMS mice showed anhedonia, whereas male CUMS mice showed more anxiogenic phenotype and social withdrawal, giving us a first glimpse on sex differences in response to chronic stress and facial validation of our CUMS protocol.

Later, we examined how different glial cell types, namely microglia, MHCII^+^ microglia, OPCs, and astrocytes, responded to stress in the hippocampi of CUMS mice. The expression of MHCII is low in microglia in homeostatic conditions but can be rapidly upregulated and is therefore often used as a marker of microglial activation and neuroinflammation in pathological conditions (Wyss-Coray and Mucke, 2002). In macrophages, MHCII has not only been shown to be enriched in M1 type but also expressed by M2b type that more closely resembles M1 macrophages than other M2 subtypes (Edwards et al., 2006). However, whether this expression pattern of MHCII can be directly extrapolated into microglial subtypes is uncertain as the definition of M1/M2 microglia *in vivo* has been objected by many researchers (Ransohoff, 2016). It should also be noted that increase of MHCII^+^ microglia may reflect an adaptive and beneficial response of microglia to stress. In fact, MHCII-expressing microglia were suggested to have neuroprotective role in some inflammatory and neurological conditions (Butovsky et al., 2005; Thored et al., 2009), which is worthy to be examined in psychiatric disorders more carefully.

Stress has a significant effect on neuroplasticity and behavior, staging especially the hippocampal region of the brain (Kim and Diamond, 2002). Again, we observed sex effect, as microglia, particularly the MHCII^+^ subtype of microglia, were increased only in female mice after CUMS, suggesting their increased neuroinflammatory response to stress. By contrast, decreases in percentages of MHCII^+^ microglia and OPCs in CUMS male mice compared to controls suggest immunosuppression by stress possibly *via* glucocorticoids. Sex difference in microglial activation involving the hippocampus has been documented (Nelson et al., 2019). For instance, a previous study observed more elevated corticosterone level and enhanced pro-inflammatory response in the hippocampus of female mice compared to male mice after CUMS (Liu et al., 2019). Increased microglia in female mice could be mediated by their glucocorticoid receptors, which are known to cause neuroinflammation that exacerbates anxiety and depression (Frank et al., 2012; Johnson et al., 2019). Corroboratively, glucocorticoid receptors and associated signaling genes were reported to be differentially expressed in female human subjects and rodents after stress (Miller et al., 2014; Bekhbat et al., 2018). Furthermore, female sex hormones, such as estrogens and progesterone, brought about sex differences in behavior due to stress (ter Horst et al., 2012). Our findings are also in line with a previous work showing that CUMS animals exhibited reduced proliferation of oligodendrocytes and endothelial cells in male rodents (Banasr et al., 2007).

Microglia are known to regulate neurogenesis in neurogenic niches including the hippocampal dentate gyrus (Tay et al., 2017). An earlier study showed that neurogenesis in the adult hippocampal dentate gyrus conferred an important mechanism for stress resilience (Anacker et al., 2018). Stimulation of hippocampal microglial proliferation was previously found to partially or completely reverse the depressive-like behavior and increase hippocampal neurogenesis after CUMS (Kreisel et al., 2014). Moreover, a recent study showed that decreasing Arg1^+^ microglia in the hippocampus by knocking down the microglial IL4R suppressed hippocampal neurogenesis and enhanced vulnerability to CUMS, whereas increasing Arg1^+^ microglia in the hippocampus by enhancing IL4 signaling restored hippocampal neurogenesis and the resilience to stress-induced depression *via* brain-derived neurotropic factor (BDNF) (Zhang et al., 2021). Activated microglia along with impaired adult hippocampal neurogenesis during chronic stress may hence jointly contribute to the pathophysiology underlying stress susceptibility in psychiatric disorders (Madore et al., 2020; Gomes-Leal, 2021). Whether microglial regulation of adult hippocampal neurogenesis in stress susceptibility or resilience is sex-dependent warrants future research.

Sex-specific stress response was also found at gene levels, as SARS-CoV-2 receptors *Ace2* and *Bsg* showed significant increases only in CUMS female mice, but not in male mice. *Bsg* is expressed by glial cells according to several public transcriptomic databases. Corroboratively, gender-dependent responses of SARS-CoV-2 receptors expressed in monocytes of human subjects enduring chronic stress were found in a GEO dataset as well, showing a significant increase of *FURIN* in stressed women but a decrease of *NRP1* in stressed men. Monocytes and monocyte-derived macrophages share myeloid features with activated microglia, and furthermore are postulated to contribute to various psychiatric disorders (Prinz and Priller, 2014; McKim et al., 2018), which was why we chose this human GEO dataset to compare to our findings in the rodent model. Our findings altogether suggest that immune cells may be more vulnerable to SARS-CoV-2 infection after being primed by stress and support recent clinical observations that women were more susceptible to the pandemic-induced anxiety and depression (Hou et al., 2020; Thibaut and van Wijngaarden-Cremers, 2020).

However, although we observed increase of MHCII^+^ microglia in stressed female mice, indicating enhanced microglia-mediated neuroinflammation, we did not see significant sex-dependent changes in cytokines *Il1b* and *TNF* after CUMS, which may require a big sample size for reliable detection and hence was a limitation in our experiment. Nevertheless, we found *Il7* was reduced in male CUMS mice. IL-7 is known to be vital for T-cell homeostasis and autoimmune inflammatory condition (Lawson et al., 2015) and was reported to be upregulated in patients with COVID-19 with neurological conditions (Huang et al., 2020; Pacheco-Herrero et al., 2021), but its importance for neuroinflammation and glial activation needs to be confirmed still.

The spatial distribution and glial expression of SARS-CoV-2 viral receptors in the human and mouse brains were noteworthy too. *Ace2* was shown to be expressed in the olfactory area of the human brain and in pericytes and endothelial cells (Fodoulian et al., 2020; Chen et al., 2021). Here, multiple viral receptors were seen enriched in the hippocampus and the orbital, anterior cingulate and prelimbic areas of the PFC in the mouse brain. Both the hippocampus and the PFC are known key regulators for executive functions associated with social cognition, mood regulation, decision-making, etc. (Bicks et al., 2015) and are the most sensitive brain regions to stress-induced epigenetic and transcriptomic changes (Hervé et al., 2017; Musaelyan et al., 2020). This suggests the potential sensitivities of these regions for viral entry or residence in the brain. However, it should be cautioned that supportive evidence on brain invasion of the SARS-CoV-2 virus is currently sparse. Furthermore, both its entry route and cellular host in the brain are still a mystery, with many postulations that lack solid experimental evidence to support (Desforges et al., 2019; Boldrini et al., 2021). In this regard, knowledge on differential expressions of these viral receptors among brain cell types is valuable for hypothesis-driven research, and the possible roles of glial cells in reception and propagation of the virus in the brain due to stress should be investigated more carefully in the future.

It should be noted that our preliminary study has many limitations. First of all, we did not provide any direct evidence demonstrating that stress and microglial activation would contribute to actual viral infection in the brain. Our correlational observations, therefore, need to be proved or disproved in a viral infection model. Second, we had small animal numbers in our experiments, which may cause biases in our data analysis and interpretation. Third, due to the high demand of tissue size in our flow cytometric experiment, we needed to use both hemispheric hippocampal tissues of each mouse and hence were unable to analyze gene expressions by qPCR in this region as well, which limited our understanding of region-dependent changes in viral receptors and cytokines in the mouse brain after stress. Therefore, more vigorous future studies are needed to examine whether there is a truly sex-dependent susceptibility of COVID-19 and associated neuroinflammatory responses post chronic stress.

## Concluding Remarks

Our findings provided evidence showing detrimental effects of chronic stress on brain and behavior and implied potential sex-dependent susceptibility to SARS-CoV-2 infection after chronic stress.

## Data Availability Statement

The original contributions presented in the study are included in the article/Supplementary Material, further inquiries can be directed to the corresponding author/s.

## Ethics Statement

The animal study was reviewed and approved by Estonian National Board of Animal Experiments.

## Author Contributions

LY designed and performed all the CUMS animal experiments, made the data analysis, and prepared the figures. MJ helped with the behavioral tests in CUMS, analyzed the transcriptomic data, prepared the figures, and wrote the manuscript. KC did the flow cytometry experiment in CUMS. AZ critically analyzed and improved the manuscript. LT supervised the project and CUMS experiments, analyzed the human transcriptomic data, and wrote the manuscript. All authors contributed to the article and approved the submitted version.

## Conflict of Interest

The authors declare that the research was conducted in the absence of any commercial or financial relationships that could be construed as a potential conflict of interest.

## Publisher’s Note

All claims expressed in this article are solely those of the authors and do not necessarily represent those of their affiliated organizations, or those of the publisher, the editors and the reviewers. Any product that may be evaluated in this article, or claim that may be made by its manufacturer, is not guaranteed or endorsed by the publisher.

## Footnotes

1 https://mouse.brain-map.org/gene/show/11210,/45849,/17953,/80514091,/18316,/12001.
