# Supplementary material for: Sex-Specific Microglial Activation and SARS-CoV-2 Receptor Expression Induced by Chronic Unpredictable Stress
Source: Front Cell Neurosci. 2021 Nov 24;15:750373. doi: 10.3389/fncel.2021.750373 (PMC8654352; doi:10.3389/fncel.2021.750373)
Supplement: Supplementary file 1 [file Data_Sheet_1.pdf]

Supplementary figure 1. **Behavioral changes after CUMS.** (**A**) Bodyweight measurements of female and male mice during 5 weeks of CUMS treatment. (**B**) Total distance moved and (**C**) ratio of time spent in corners versus center in open field test. CUMS had main effect on open field corner/center ratio (**C**, F (1, 15) =7.29, $p < 0.05$, 2-way ANOVA).

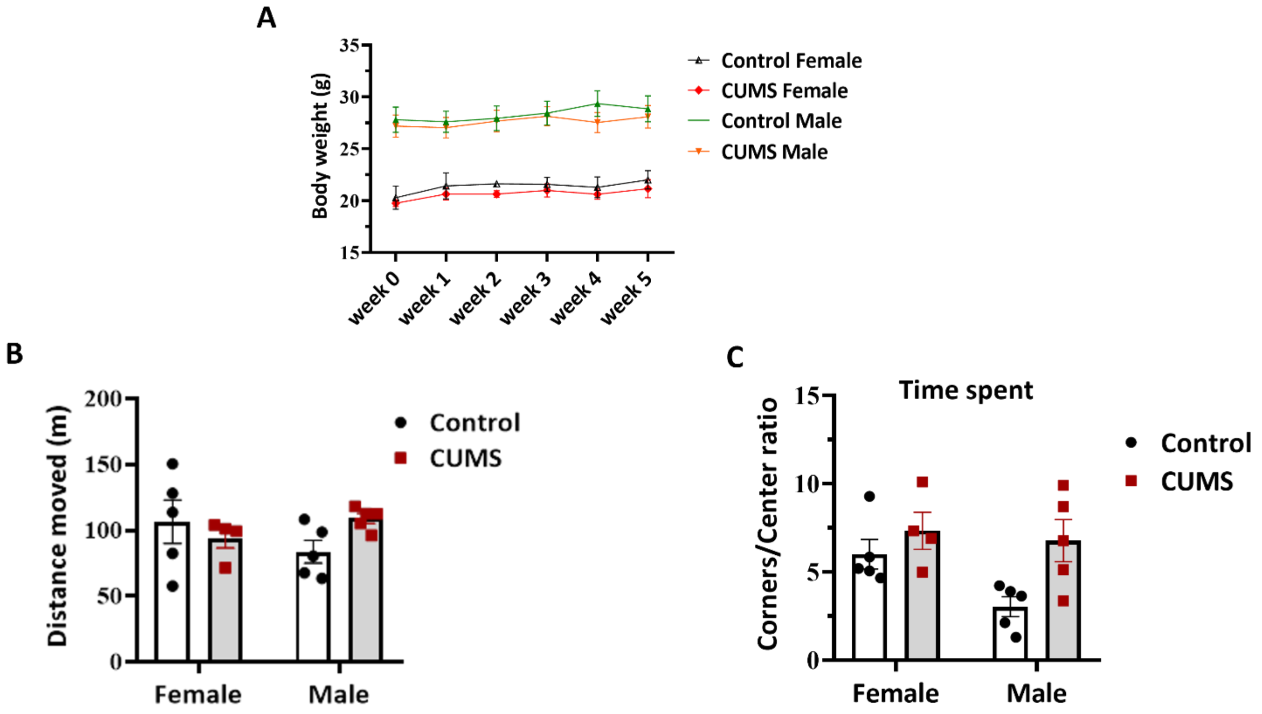

Supplementary figure 2. **Percentages of astrocytes and OPCs in the hippocampus.** Representative dot plots of isotype control antibodies **(A)** used in flow cytometric analysis and cell-specific staining for astrocytes and OPCs (**B**) are shown. Percentage of astrocytes **(C)** and percentage of OPCs **(D)** present among total brain cells present in the hippocampus. CUMS main effect on OPCs (**D**, F (1, 15) = 9.44, $p < 0.01$) and sex main effect on astrocytes (**C**, F (1, 15) = 8.5, $p < 0.01$, 2-way ANOVA) were observed, respectively.

A

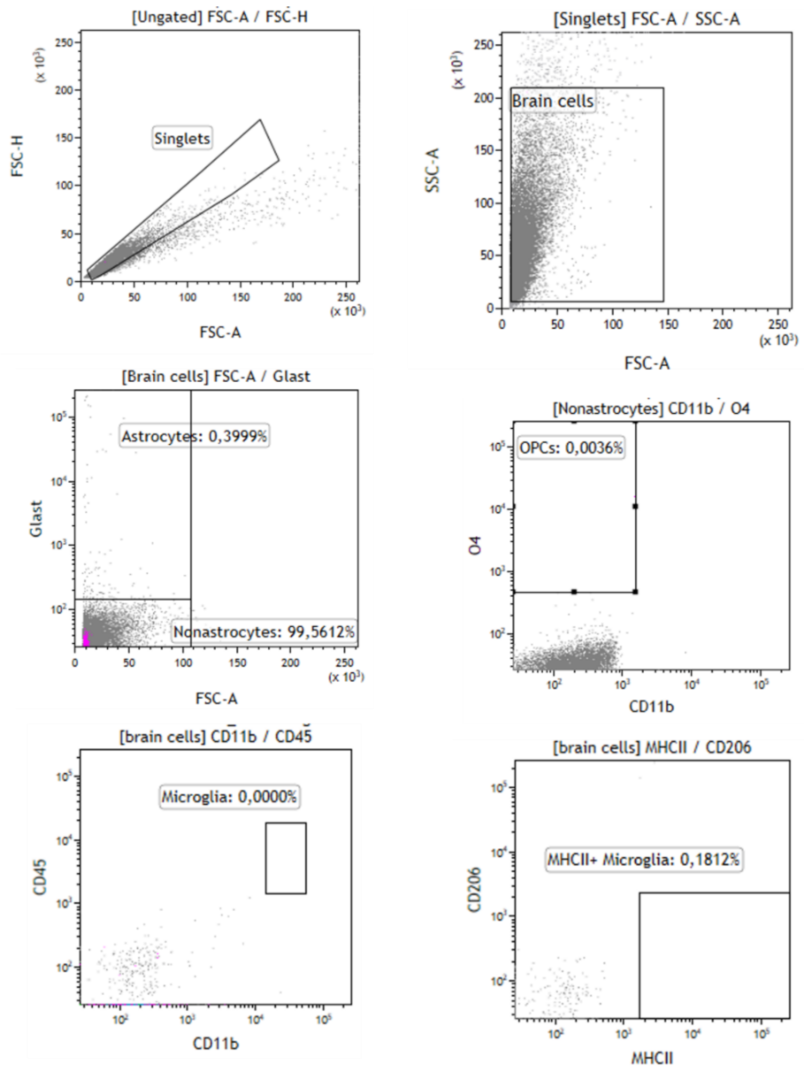

B

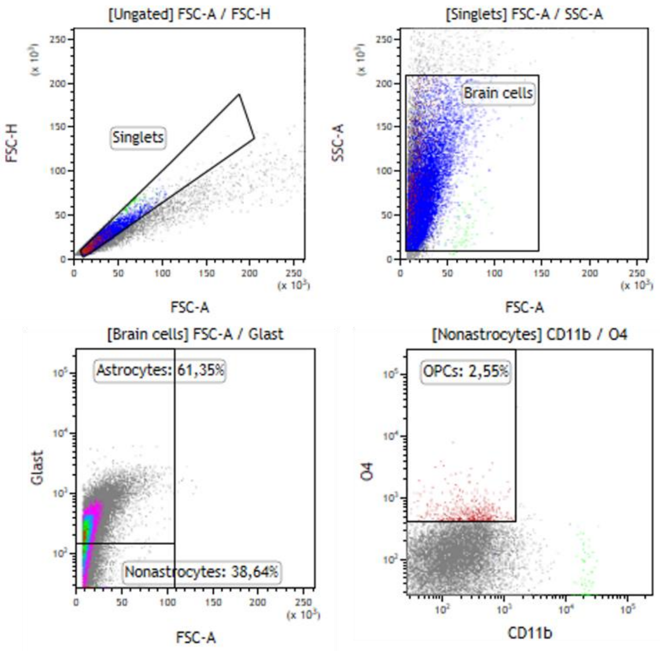
[Ungated] FSC-A / FSC-H
Singlets
FSC-H
FSC-A
[Singlets] FSC-A / SSC-A
Brain cells
SSC-A
FSC-A
[Brain cells] FSC-A / Glast
Astrocytes: 61,35%
Nonastrocytes: 38,64%
Glast
FSC-A
[Nonastrocytes] CD11b / O4
OPCs: 2,55%
O4
CD11b

C

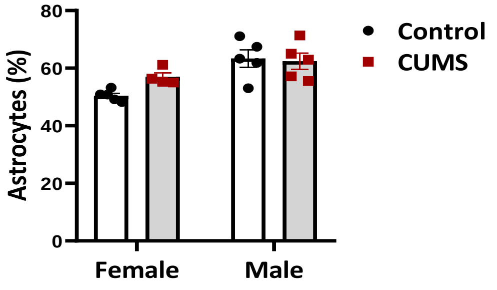
Astrocytes (%)
80
60
40
20
0
Female
Male
Control
CUMS

D

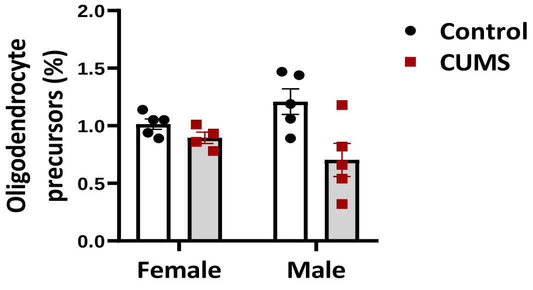
Oligodendrocyte precursors (%)
2.0
1.5
1.0
0.5
0.0
Female
Male
Control
CUMS

Supplementary figure 3. Human Protein Atlas (HPA) dataset. Brain expression data obtained through RNA-seq generated reported as normalized expression (NX) for human prefrontal cortex samples. Color coding of the bars separates prefrontal cortex and the three reference cortex regions (frontal, parietal and temporal) (https://www.proteinatlas.org/).

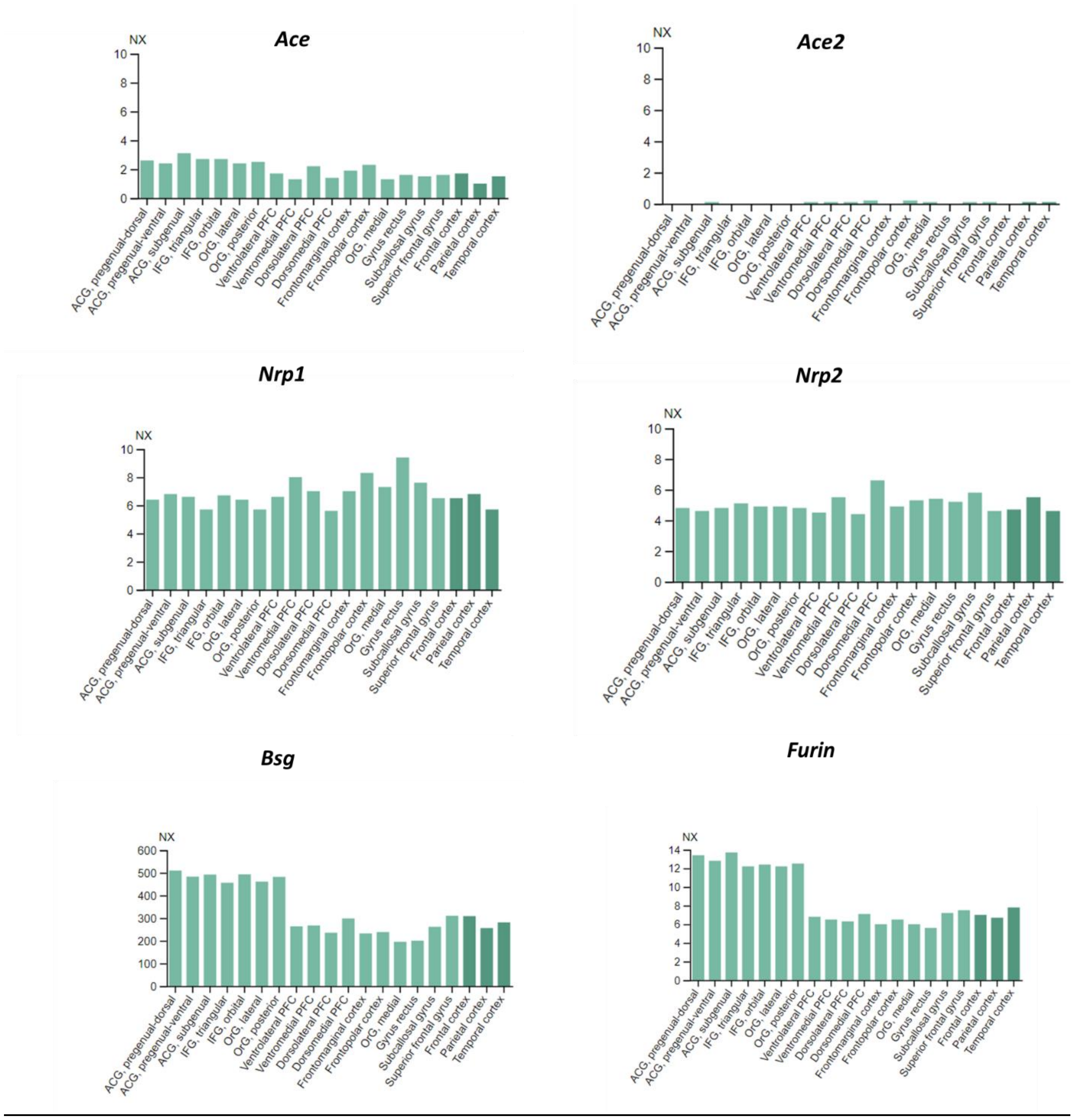

Supplementary figure 4. Brain Rnaseq database from Barres's lab (https://www.brainrnaseq.org/) was used for checking cell-specific expressions of *Ace, Ace2, Nrp1, Nrp2, Bsg* and *Furin* genes in the normal mouse and human brains.

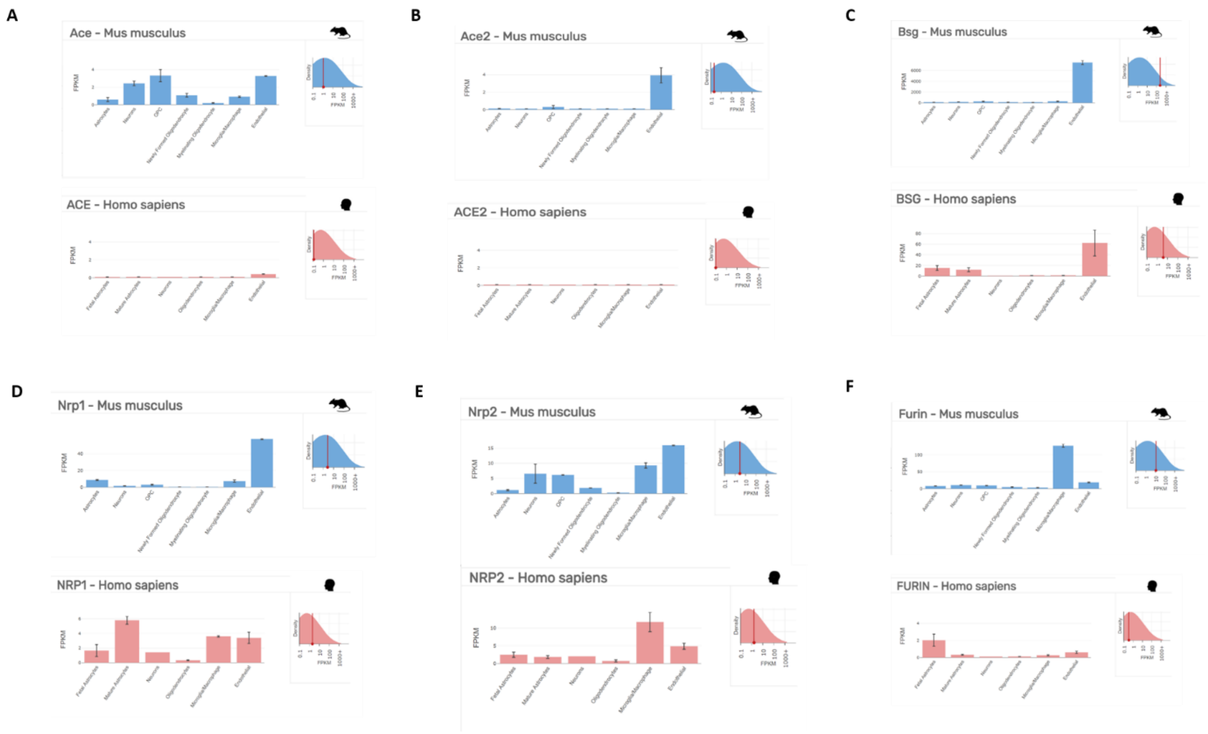

Supplementary figure 5. Brain vascular single-cell database from Belsholtz lab (http://betsholtzlab.org/VascularSingleCells/database.html) was used for cell type distrubution of *Ace, Ace2, Nrp1, Nrp2, Bsg* and *Furin* genes in the mouse brain. The different cells are namely Pericytes, venous SMC, arteriolar SMC, arterial SMC, Microglia, fibroblast-like type 1, fibroblast-like type 2, Oligodendrocytes, EC type 1, EC type 2, EC type 3, venous EC, capillary EC, arterial EC and Astrocyte. The abbreviations are PC - Pericytes; SMC - Smooth muscle cells; MG - Microglia; FB - Vascular fibroblast-like cells; OL - Oligodendrocytes; EC - Endothelial cells; AC - Astrocytes; v - venous; capil - capillary; a - arterial; aa - arteriolar; 1,2,3- subtypes.

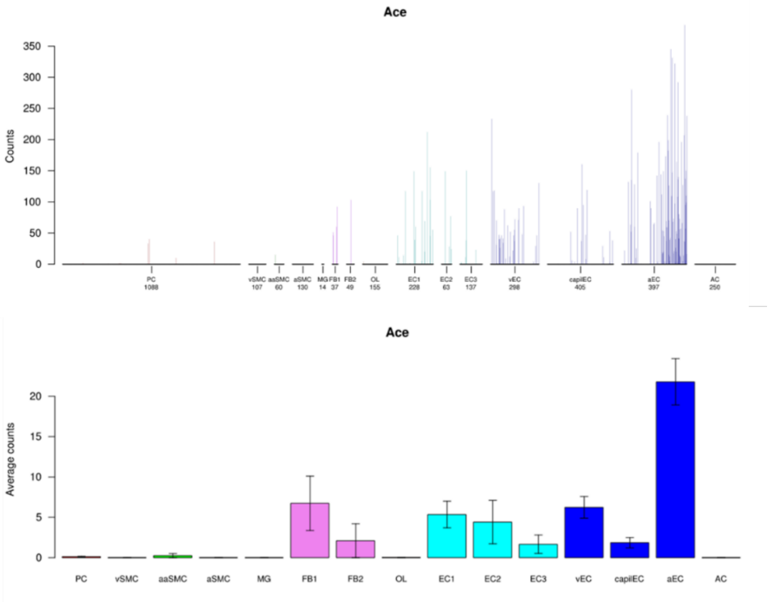

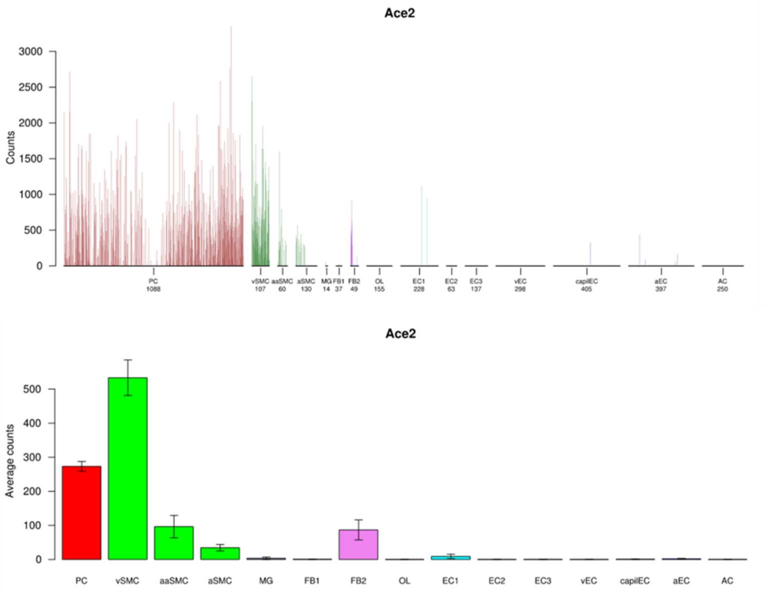

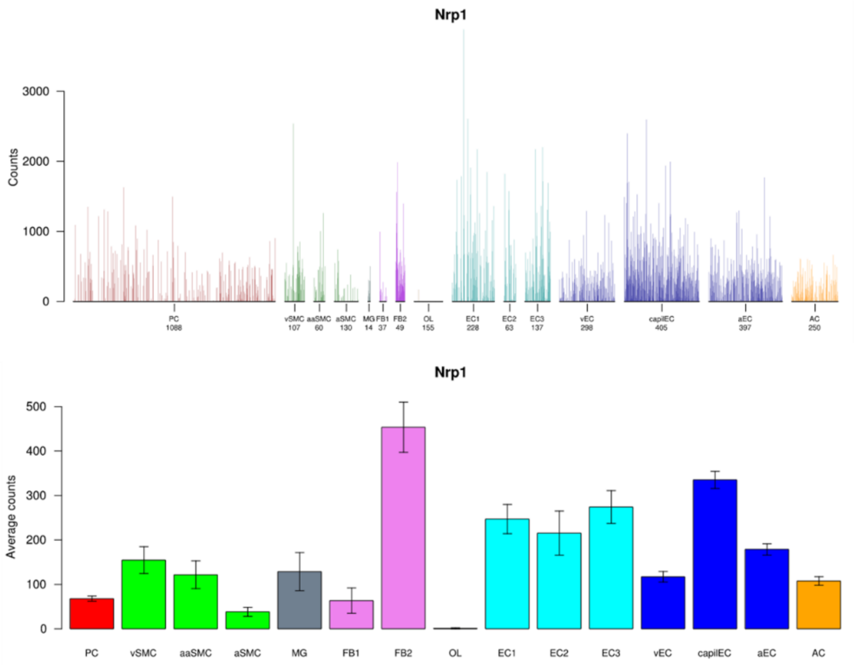
Nrp1
Counts
3000
2000
1000
0
PC 1088
vSMC 107
aaSMC 60
aSMC 130
MG 14
FB1 37
FB2 49
OL 155
EC1 228
EC2 63
EC3 137
vEC 298
capilEC 405
aEC 397
AC 250
Nrp1
Average counts
500
400
300
200
100
0
PC
vSMC
aaSMC
aSMC
MG
FB1
FB2
OL
EC1
EC2
EC3
vEC
capilEC
aEC
AC

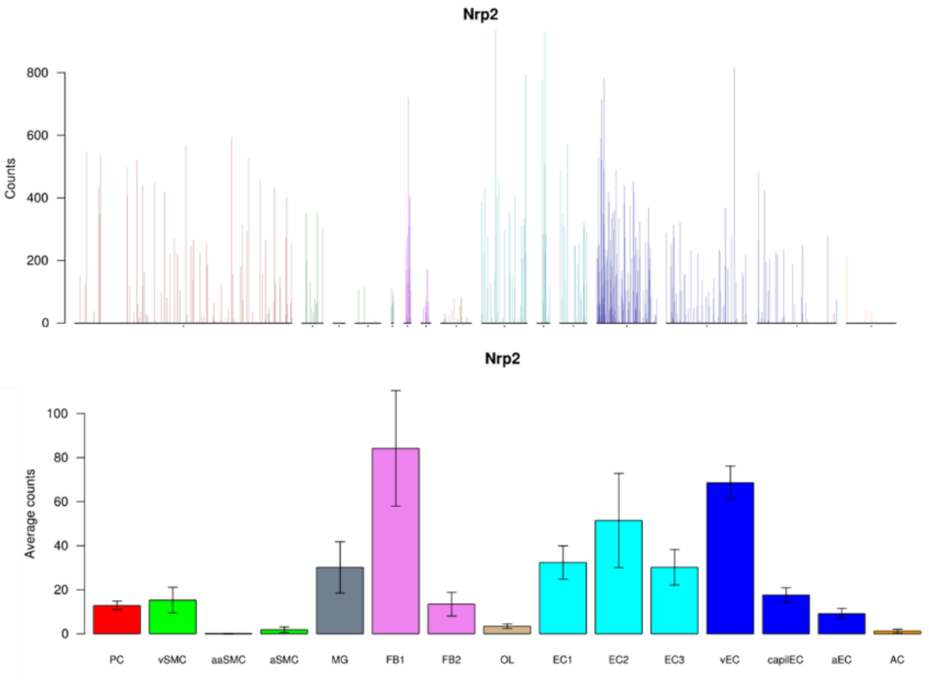
Nrp2
Counts
800
600
400
200
0
Nrp2
Average counts
100
80
60
40
20
0
PC
vSMC
aaSMC
aSMC
MG
FB1
FB2
OL
EC1
EC2
EC3
vEC
capilEC
aEC
AC

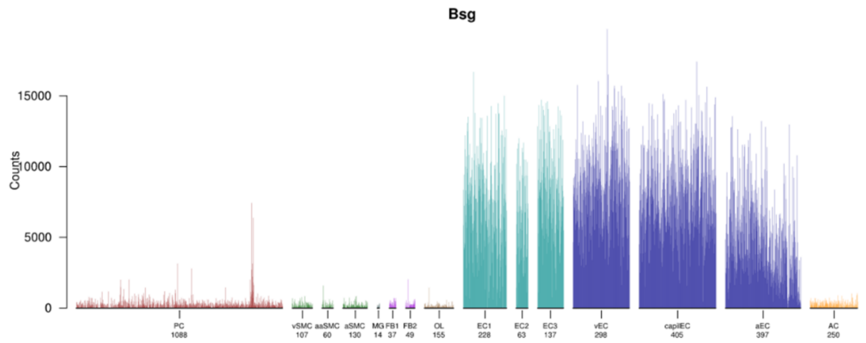
Bsg
Counts
15000
10000
5000
0
PC 1088
vSMC 107
aaSMC 60
aSMC 130
MG 14
FB1 37
FB2 49
OL 155
EC1 228
EC2 63
EC3 137
vEC 298
capilEC 405
aEC 397
AC 250

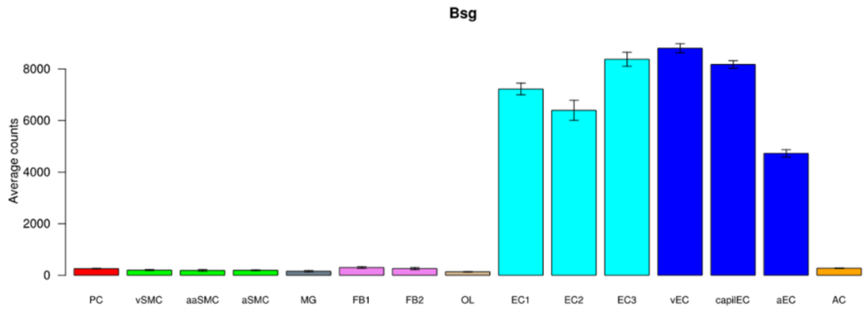
Bsg
Average counts
8000
6000
4000
2000
0
PC
vSMC
aaSMC
aSMC
MG
FB1
FB2
OL
EC1
EC2
EC3
vEC
capilEC
aEC
AC

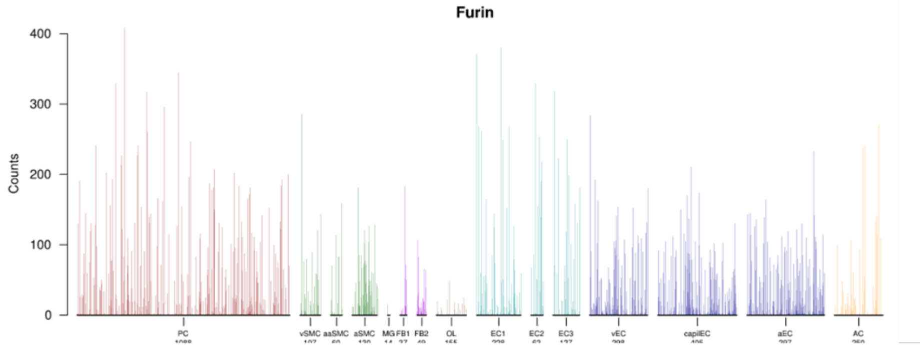
Furin
Counts
400
300
200
100
0
PC
vSMC
aaSMC
aSMC
MG
FB1
FB2
OL
EC1
EC2
EC3
vEC
capilEC
aEC
AC

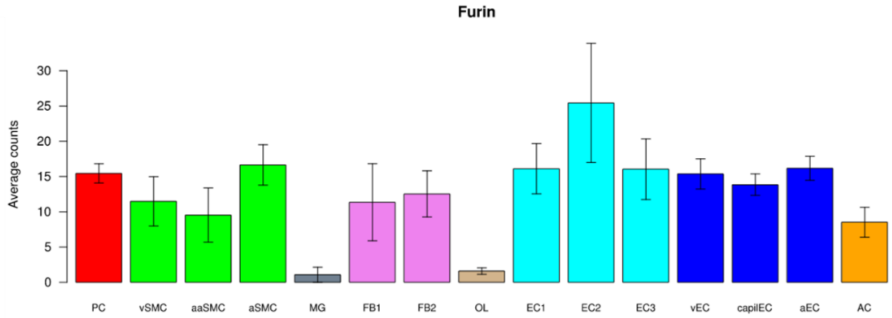
Furin
Average counts
30
25
20
15
10
5
0
PC
vSMC
aaSMC
aSMC
MG
FB1
FB2
OL
EC1
EC2
EC3
vEC
capilEC
aEC
AC

Supplementary figure 6. Single-cell RNA sequencing data of *Ace, Ace2, Nrp1, Nrp2, Bsg* and *Furin* genes in different cell subtypes in the mouse prefrontal cortex (http://djeknad.pythonanywhere.com/, Bhattacherjee et al., 2019). Representative Track plots for all the 6 genes in different cell types are given below.

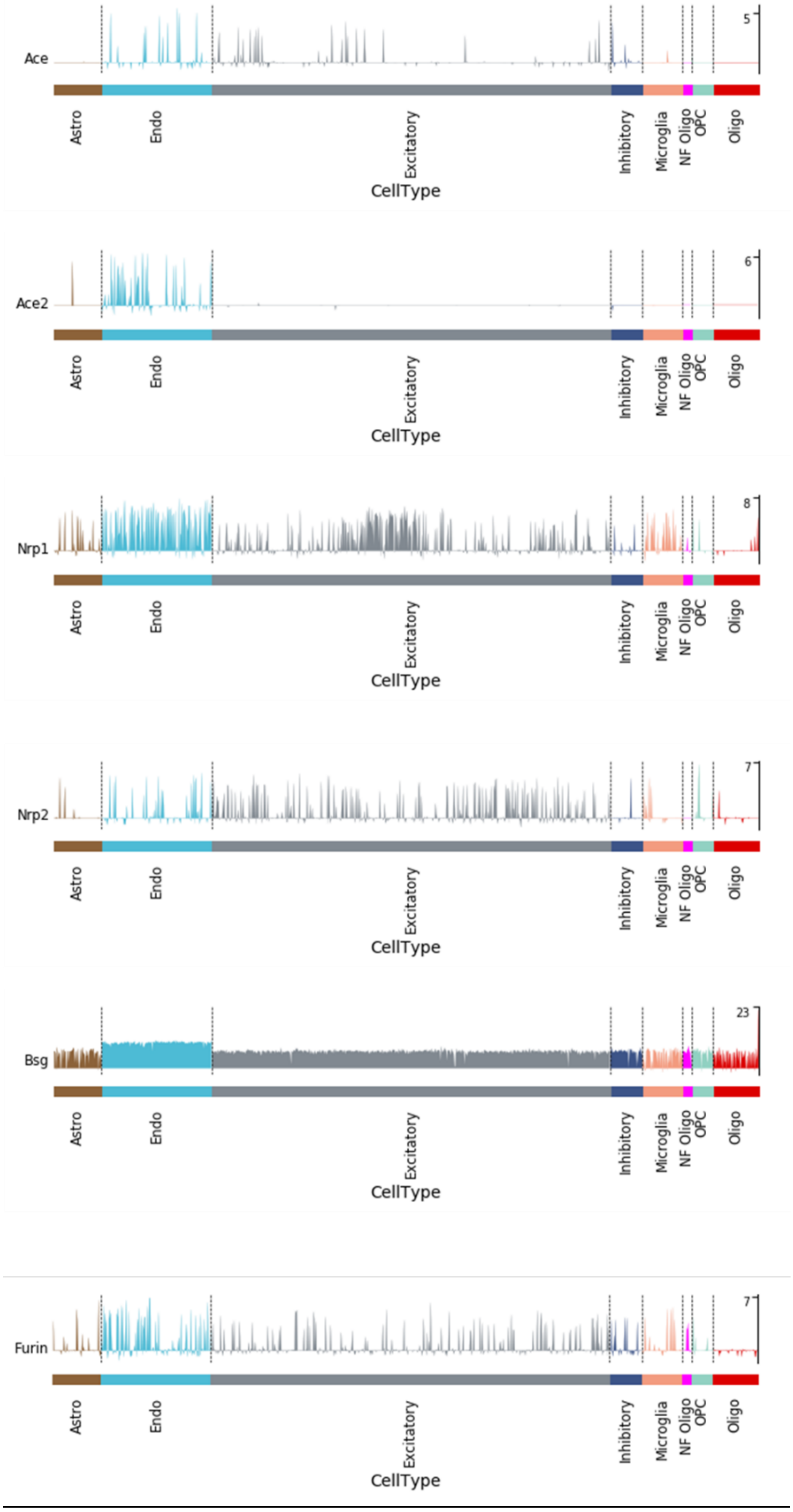

<u>Supplementary figure 7:</u> SARS CoV2 viral receptors and pro-inflammatory cytokine genes expressed in the female and male brains. *Ace, Ace2b, Nrp1, Nrp2, Furin, Tnf*, *Il7 and Il1b* mRNA expressions were studied using RT-QPCR. CUMS main effects on *Ace2b* (**B**, F (1, 15) = 9.37, *p* < 0.01) and *Il7* (**G**, F (1, 15) = 11.77, *p* < 0.01) and sex main effect on *Nrp1* (**C**, F (1, 15) = 8.00, *p* < 0.05) were observed, respectively (2-way ANOVA).

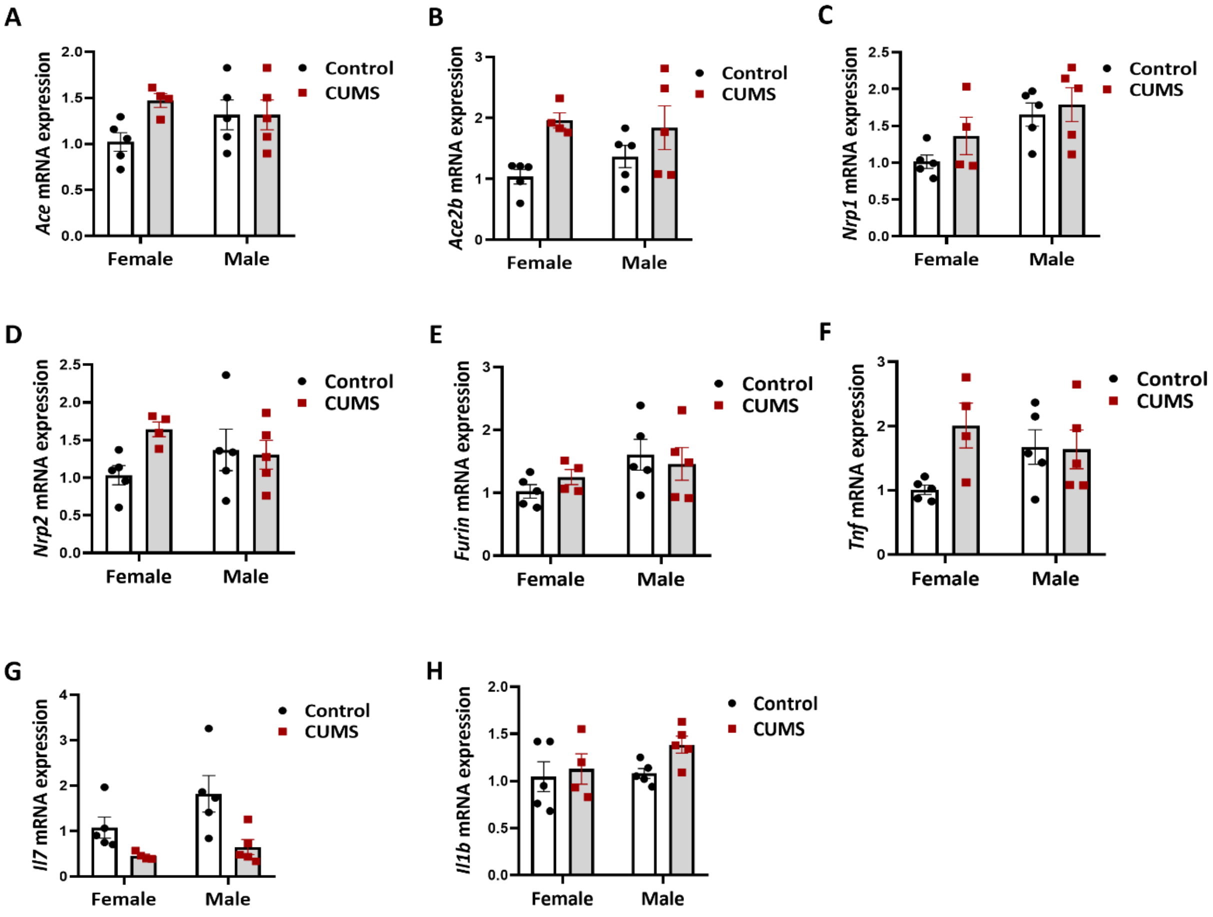

Supplementary table 1. Primers designed for *Ace, Ace2, Nrp1, Nrp2, Bsg* and *Furin* genes for expression studies in mice.

Gene name	Primer Sequence 5'-3'	
Ace2a	**Forward**	CTACAGGCCCTTCAGCAAAG
	**Reverse**	TGCCCAGAGCCTAGAGTTGT
Ace2b*	**Forward**	TCCATTGGTCTTCTGCCATCC
	**Reverse**	AACGATCTCCCGCTTCATCTC
Ace	**Forward**	CCACTGACAGAATGGCTCGT
	**Reverse**	GTGGGTGTAGTACCGGTGTTT
Bsg	**Forward**	ACTGGGGAAGAAGAGGCAATC
	**Reverse**	AACCAACACCAGGACCTCAG
Nrp1	**Forward**	GGAGCTACTGGGCTGTGAAG
	**Reverse**	ACCGTATGTCGGGAACTCTG
Nrp2	**Forward**	CTGGTTAGTAGCCGCTCTGG
	**Reverse**	TCCCAGTCCTTGCCATTTAG
Furin	**Forward**	CATTCGTATGGCTACGGGCT
	**Reverse**	GGGCTGATGAGGTGGATAGC
Il7	**Forward**	CTAAATCGTGCTGCTCGCAA
	**Reverse**	TTCACCAGTGTTTGTGTGCC
Tnf	**Forward**	ATGGCCTCCCTCTCATCAGT
	**Reverse**	TTTGCTACGACGTGGGCTAC
Il1b	**Forward**	TGGACCTTCCAGGATGAGGACA
	**Reverse**	GTTCATCTCGGAGCCTGTAGTG
β-actin	**Forward**	ACTGAGCTGCGTTTTACACCC
	**Reverse**	GCCTTCACCGTTCCAGTTTT

*published primers from Ma et al., 2020.

Supplementary table 2. Spatial distribution of these genes in the frontal cortex brain areas namely the anterior cingulate cortex, prelimbic area, infralimbic area and the orbital area of mice.

Frontal cortex brain areas				
	**Anterior cingulate cortex**	**Prelimbic area**	**Infralimbic area**	**Orbital area**
Ace	-	-	-	Orbital Area, ventral part, layer 2/3
	-	-	-	Orbital area, lateral part, layer 6a
	-	-	-	Orbital area, medial part, layer 5
Ace2	Anterior cingulate area, dorsal part, layer 6a	Prelimbic area, layer 5	-	-
Nrp1	-	-	-	-
Nrp2	-	-	Infra limbic, layer 2	Orbital area, lateral part, layer 2/3
	-	-	Infra limbic, layer 2/3	Orbital area, lateral part, layer 5
	-	-	Infra limbic, layer 5	Orbital area, ventrolateral part, layer 5
	-	-	Infra limbic, layer 6b	Orbital area, ventrolateral layer 2/3
	-	-		Orbital area, lateral part, layer 6a
	-	-		Orbital area, lateral part, layer 6b
	-	-		Orbital area, ventrolateral layer 6b
Bsg	Anterior cingulate area, Ventral part, layer 5	Prelimbic area, layer 2	-	Orbital area, medial part, layer 2/3
	Anterior cingulate area, Ventral part, layer 6a	Prelimbic area, layer 5	-	Orbital area, lateral part, layer 6a
	Anterior cingulate area, Ventral part, layer 2/3	Prelimbic area, layer 2/3	-	Orbital area, medial part, layer 6a
	Anterior cingulate area, Dorsal part, layer 5		-	Orbital area, lateral part, layer 5
	Anterior cingulate area, Dorsal part, layer 2/3		-	Orbital area, medial part, layer 2
			-	Orbital area, medial part, layer 5

			-	Orbital area, ventrolateral part, layer 5
			-	Orbital area, ventrolateral part, layer 2/3
			-	Orbital area, lateral part, layer 2/3
			-	Orbital area, lateral part, layer 6b
Furin	Anterior cingulate area, Dorsal part, layer 6a	Prelimbic area layer 6a	Infralimbic layer 6b	Orbital area, medial part, layer 2/3
	Anterior cingulate area, ventral part	Prelimbic area layer 6b	Infralimbic area layer 5	Orbital area, ventrolateral part, layer 2/3
	Anterior cingulate area, dorsal part, layer 2/3	Prelimbic area layer 5	Infralimbic layer 6a	Orbital area, ventrolateral part, layer 5
	Anterior cingulate area, dorsal part, layer 5			Orbital area, ventral part, layer 6a
	Anterior cingulate area, dorsal part, layer 6b			Orbital area, medial part, layer 6a
				Orbital area, ventrolateral, part 6a
				Orbital area, medial part, layer 2
				Orbital area, ventrolateral
